# Plant Toxic Proteins: Their Biological Activities, Mechanism of Action and Removal Strategies

**DOI:** 10.3390/toxins15060356

**Published:** 2023-05-24

**Authors:** Emine Kocyigit, Betul Kocaadam-Bozkurt, Osman Bozkurt, Duygu Ağagündüz, Raffaele Capasso

**Affiliations:** 1 Department of Nutrition and Dietetics, Ordu University, Cumhuriyet Yerleşkesi, 52200 Ordu, Turkey; kocyigitem@gmail.com; 2 Department of Nutrition and Dietetics, Erzurum Technical University, Yakutiye, 25100 Erzurum, Turkey; betulkocaadam@gmail.com (B.K.-B.); dytosmanbozkurt@gmail.com (O.B.); 3 Department of Nutrition and Dietetics, Gazi University, Faculty of Health Sciences, Emek, 06490 Ankara, Turkey; duyguturkozu@gazi.edu.tr; 4 Department of Agricultural Sciences, University of Naples Federico II, 80055 Portici, Italy

**Keywords:** plant protein, toxins, biological activity, possible usage, reduction strategies

## Abstract

Plants evolve to synthesize various natural metabolites to protect themselves against threats, such as insects, predators, microorganisms, and environmental conditions (such as temperature, pH, humidity, salt, and drought). Plant-derived toxic proteins are often secondary metabolites generated by plants. These proteins, including ribosome-inactivating proteins, lectins, protease inhibitors, α-amylase inhibitors, canatoxin-like proteins and ureases, arcelins, antimicrobial peptides, and pore-forming toxins, are found in different plant parts, such as the roots, tubers, stems, fruits, buds, and foliage. Several investigations have been conducted to explore the potential applications of these plant proteins by analyzing their toxic effects and modes of action. In biomedical applications, such as crop protection, drug development, cancer therapy, and genetic engineering, toxic plant proteins have been utilized as potentially useful instruments due to their biological activities. However, these noxious metabolites can be detrimental to human health and cause problems when consumed in high amounts. This review focuses on different plant toxic proteins, their biological activities, and their mechanisms of action. Furthermore, possible usage and removal strategies for these proteins are discussed.

## 1. Introduction

Plants have been used in medicine, assassination, and hunting for centuries thanks to the bioactive compounds they contain. Plants were used as a poison by the Egyptians and Romans, and they have been used as a raw material for medicines from ancient times to the present [1]. Today, the use of plant-based foods is increasing in nutritional supplements, teas, and the production of medicines. However, it is a misconception that every product obtained from plants is natural and safe [2]. Toxins can be found in the roots, leaves, fruits, and seeds of plants [3].

Plants are subject to a variety of stressors originating from their environment. Apart from these variables, there exists a persistent threat of predators and pathogens. Depending on the type of adverse conditions, plants possess different defense mechanisms; some of them produce toxic natural bioactive compounds [4]. Low molecular weight toxic compounds include alkaloids, terpenoids, tannins, and glycosides. Plants have the ability to produce toxic proteins, including lectins and ribosome-inactivating proteins (RIP), as a means of survival [5,6]. Both beneficial and detrimental impacts are exhibited by these chemicals in both humans and animals. Numerous side effects are exhibited by these substances, including, but not limited to, mild itching, vomiting, nausea, psychological disorders, palsy, embryonic malformations, and cardiac dysrhythmias. However, they also possess advantageous properties in the management of illnesses, such as cancers and peptic ulcers, as well as in industries such as pharmaceuticals and cosmetics. They can cause disease by interacting with enzymes, biological macromolecules, such as cellular receptors, or by absorption into body tissues [7,8].

Plants express a variety of toxic proteins that confer resistance against both living organisms (such as pests, insects, pathogens, and parasites) and environmental conditions (such as temperature, pH, humidity, salt, drought, and so on). Several families of toxic proteins have been identified, such as lectins, RIPs, protease inhibitors, α-amylase inhibitors, ureases, arcelins, antimicrobial peptides, and pore-forming toxins [9]. The leaves, fruits, roots, bark, and flowers of both poisonous and nonpoisonous plants contain these toxins [3,7]. Moreover, plant toxins serve as defense proteins and help in plant growth and survival [10]. The human body takes in plant toxins through the consumption of plants, physical contact with plants, and the intake of contaminated food and water. At certain exposure levels, these molecules are usually regarded as safe. Human consumption of plant toxins has grown as interest in the utilization of plant components for medications, food and feed, tea, and extracts has expanded [11,12,13].

Plant toxic proteins are a highly significant class of compounds in all sectors, including health, security, agriculture, and drug discovery, among others. Despite the fact that numerous studies have been conducted, it is essential to classify these compounds according to their origins, mechanisms of action, risks, and benefits. This study reviewed plant toxic proteins in edible plants and their biological activities, possible usage, and reduction strategies.

## 2. Plant Toxic Proteins

### 2.1. Ribosome İnactivating Proteins

Ribosome inactivating proteins (RIPs) are cytotoxic enzymes, first discovered in castor oil (Ricinus communis) predominantly produced by plants and some bacteria [14]. Ribosome inactivating proteins exhibit rRNA N-glycosidase activity (EC 3.2.2.22). This is accomplished by the enzymatic removal of an adenine residue (A-4324) located on the 28S RNA of the large ribosomal subunit 60S [15,16]. Many RIPs are produced by plants, including ricin, abrin, and saporins, as a defensive strategy against viral or parasitic invaders. Others, such as the Shiga toxins, are generated by pathogenic bacteria as virulence factors to help in their reproduction and survival in host species [17,18]. Once produced, RIPs, which are strong cellular poisons, are typically exported from the cell and localized inside the cell wall matrix. As the pathogen penetrates the cell, it is thought that it obtains access to the cytoplasm, therefore enhancing its activity by inhibiting host ribosomes [19]. Several plant families are particularly rich in RIPs; among them are the Cucurbitaceae, Caryophyllaceae, Phytolaccaceae, Poaceae, Euphorbiaceae, and Nyctaginaceae families. They are very abundant in seeds and fruits yet low in leaves and stems. Isoforms of RIPs may coexist in one organ or occur in different organs [20,21,22].

Over a hundred distinct plant species have been identified with RIPs, and they may be found in a variety of plant organs. RIPs are classified into three types based on the structure of their protein domains [23,24]. Type I RIPs are single-chain proteins with a molecular weight of approximately 30 kDa that possess RNA N-glycosidase enzymatic activity, such as pokeweed antiviral protein (PAP), trichosanthin (TCS), and saporin (SO6) [25]. Type II RIPs, such as ricin and abrin, have an A-chain with RNA N-glycosidase activity and one or more B-chains linked by a disulfide bridge. The B-chain is a lectin-like peptide with a high affinity for galactose residues on cell surfaces that facilitates translocation through the plasma membrane, thus the B-chain enables the A-chain to enter the cell [26]. Type III RIPs comprise an amino-terminal domain similar to type I RIPs with a carboxy-terminal region with uncertain function; examples are barley JIP60 16 and maize ribosome-inactivating protein b32 [27]. The maize protein, b32, is synthesized as an inactive proenzyme that is activated after an internal peptide fragment is removed, producing the N-terminal and C-terminal prosequences that appear to function together as an N-glycosidase. JIP60 is made up of an amino group-terminal domain that is comparable to type 1 RIPs and a carboxyl-terminal domain that is similar to eukaryotic translation initiation factor 4E. Because of their distinct structures, these two proteins cannot be classified as type 1 RIPs. All kinds of RIP suppress protein synthesis through a variety of distinct mechanisms [24,28,29].

In most cases, RIPs are responsible for the removal of a particular adenine located in the α-sarcin/ricin loop (α-SRL) of rRNA. This results in the inhibition of the binding of elongation factor. Because the α-SRL loop has been depurinated, the GTP binding site has lost its capacity to stimulate GTP hydrolysis. Therefore, protein synthesis is inhibited [30,31]. Due to their lectin-binding capabilities, the majority of type 2 RIPs exhibit a greater rate of cell entrance and, thus, cytotoxicity. Furthermore, RIPs show polynucleotide adenine glycosylase (PAG) activity on a variety of nucleic acid substrates [23,32]. In addition to superoxide dismutase, deoxyribonuclease, chitinase, and lipase activity, RIPs have been reported to possess various enzymatic activities [33,34].

Due to their various antibacterial, antifungal, and insecticidal characteristics, plant RIPs are used as traditional natural antibiotics [34]. RIPs have an antiviral effect by inhibiting protein synthesis in virus-infected cells. This suggests a function for RIPs in antiviral treatments [35,36]. It is thought that viral infection facilitates the entrance of RIP, which then inactivates cell ribosomes, causing cell death and preventing the virus from reproducing and spreading. Furthermore, all RIPs release adenines from eukaryotic DNA, and many also release adenines from other RNAs, such as viral RNAs. Some RIPs have DNA-nicking, DNase, or RNAse activity, which can disrupt viral replication, transcription, translation, and assembly [37,38]. Evidence suggests that RIPs, which have PAG activity on viral RNAs, inhibit the translation of capped RNA by binding to the cap of viral RNAs and depurinating them downstream of the cap structure. PAP may also bind to translation-initiating proteins, allowing it to depurify uncapped viral RNAs selectively [39,40].

T-cells infected with HIV are able to activate a recombinant form of maize-RIP proenzyme by adding an HIV protease recognition sequence between the pro-peptide and active RIP [41]. Similarly, PAP has been shown to be effective against a broad variety of viruses, including HIV, herpes simplex virus, cytomegalovirus, influenza virus, polio virus, hepatitis B virus, and DNA virus [42,43]. Clinical trials of TCS in AIDS patients who have not responded to zidovudine have shown a considerable increase in circulating CD4^+^ T cells and a substantial decrease in p24 levels [44]. When MAP30 is exposed to HepG cells, HBV DNA replication and HBsAg secretion are inhibited. In addition, the expression of the HBV antigen is suppressed by MAP30, viral DNA replication is downregulated, replicative intermediates are downregulated, and cDNA synthesis is reduced [45]. It has also been hypothesized that RIPs may exert their antiviral effects via signaling pathways. During viral infection, RIPs have been found to increase p53 and c-Jun N-terminal kinase (JNK) activity while suppressing KF-B, p38MAPK, and Bcl-2 activation. The regulation of these pathways would lead to the death of infected cells, hence preventing the spread of the virus [46].

In addition to their glycosidase activity, RIPs possess antitumor, anticancer, antiviral, abortifacient, and neurotoxic activities. Several RIPs, including TCS, α-momorcharin, MAP30, abrin, and ricin, have been shown to trigger apoptosis in cancer cells, hence inhibiting the proliferation of tumor cells in numerous forms of cancer, including breast cancer, leukemia/lymphoma, and hepatoma. As a result of changes in receptor concentration on malignant cell surfaces or the altered intracellular transit of toxins, cancer cells are more vulnerable to the toxicity of RIPs than healthy cells [34,47]. TCS, which is derived from the Trichosanthes kirilowii plant, is used in TCM for both inducing abortion and treating hydatidiform lesions [48]. 

### 2.2. Lectins

Lectins are glycan-binding proteins containing carbohydrate-binding sites that enable them to recognize and bind certain carbohydrate structures (i.e., monosaccharides and oligosaccharides) via hydrogen-bonded and hydrophobic interactions [49,50]. The carbohydrate-binding capacities of lectins determine their biological roles, such as immunological responses, cell–cell interactions, signaling pathways, and cell growth. Lectins uniquely recognize and reversibly bind to carbohydrates. Lectins identify the monosaccharides glucose, galactose, fucose, and mannose. N- and O-linked oligosaccharides are the primary attachment sites for the majority of glycans [51]. Most lectins have been discovered in plants, but they have also been found in mammals, insects, viruses, fungi, and bacteria [52,53]. Lectins may interact with both water and carbohydrates, and they can bind to metal ions [54]. 

First discovered in plants (ricin from *Ricinus communis* and abrin from *Abrus precatorius*), lectins have now been discovered in a wide range of organisms. These lectins exhibit the ability to agglutinate blood cells and possess the property of RIPs. Landsteiner and Raubitschek discovered non-toxic lectins in the legumes *Phaseolus vulgaris* (bean), *Psium sativum* (pea), *Vicia sativa* (vetch), and *Lens culinaris* (lentil), refuting the theory that all proteins with agglutinating activity are poisonous [55]. In addition, not all lectins exhibited agglutination activity, hence lectins are now categorized as carbohydrate-binding sites [56,57]. Based on the number of carbohydrate-binding sites they possess, lectins are classified as merolectins (binding with only one carbohydrate), hololectins (binding with two carbohydrate structures that are not related), chimerolectins (binding with one or more substrates and another independent catalytic domain), or superlectins (binding with multiple carbohydrate structures that are not related) [58]. 

All plants contain lectins, although the highest levels are found in uncooked legumes (beans, lentils, peas, soybeans, and peanuts), nuts, and cereals. It has been discovered that lectins serve many crucial functions in a range of biological processes [59]. In microorganisms, lectins have an important role in cell surface adhesion, which is essential for colonization, viral infections, bacteria, fungus, and symbiotic microbes associating with the host. Certain lectins are utilized as antimicrobials, antibacterial, antifungal, antiviral, and anticancer agents because they bind to carbohydrates on the surface of microorganisms, causing holes to develop, affecting cell permeability, and perhaps interacting with microbe cell wall components. Moreover, lectins can suppress microbial growth by interfering with biofilm formation and the quorum sensing process. As secondary metabolites, plants secrete lectins as a defensive strategy against several pathogens. Its resistance to digestion is one of the species’ most noticeable features. Thus, lectins may influence intestinal permeability via interactions with epithelial cells. Studies on animals have demonstrated that large dosages of isolated lectins can impact the intestinal mucosa, resulting in altered food absorption, immunological activation, and permeability [60,61]. 

In vitro and in vivo studies have demonstrated that lectins can suppress cancer cell proliferation by functioning as antiangiogenic, antimetastatic, and antiproliferative agents. This indicates that lectins may be effective in cancer therapy [62,63,64]. The potential use of lectins as anticancer medicines has been investigated in a small number of clinical studies [65,66]. The antiproliferative effects of lectins on human cancer cell lines may be attributable to their capacity to induce apoptosis and autophagy via regulating the synthesis of caspase and other proteins [59].

The antimicrobial, antibacterial, antifungal, and antiviral properties of lectins have also been detected. Lectins have several mechanisms of antibacterial activity, including the suppression of cell development, the destruction of the cell wall produced by contact with bacterial cell wall components (N-acetylmuramic acid, N acetylglucosamine, lipopolysaccharides), and the agglutination of bacterial cells. Lectins prevent microorganisms from penetrating cell membranes by interacting with glycoproteins there [67,68]. Herpes simplex, Ebola, and severe acute respiratory syndrome coronavirus 2 (SARS-CoV-2) are just some of the viruses that have had their proliferation inhibited by lectins in recent in vitro studies [69]. While the exact mechanisms are unknown, these chemicals appear to have a role either during the attachment phase of viral replication or towards the conclusion of the virus’s life cycle [60,70].

Research on the possible involvement of plant lectins in warding against metabolic diseases has also been conducted. Anti-diabetic and anti-hyperlipidemic activity were discovered when lectins were isolated from the seeds of Abrus precaterius L. and administered to mice whose diabetes had been induced with alloxan monohydrate [71]. Similarly, Bryothamnion seaforthii isolated compounds utilized in a fixed dosage administration have demonstrated hypoglycemic and hypolipidemic effects, reduced insulin resistance, and improved pancreatic β-cell activity in response to oxidative stress in streptozotocin-induced rats [72]. 

Despite lectins’ potential benefit to human health, they can present a serious risk due to their ability to act as antinutritional factors and hemagglutinins [73]. They withstand digestion enzymes quite well. This suggests that lectins may influence intestinal permeability via interactions with intestinal epithelial cells. Animal studies have demonstrated that ingesting large amounts of isolated lectins can disrupt intestinal mucosal integrity and have negative effects on nutrient absorption [61,74]. The lectin content can be reduced by the use of typical processing or cooking procedures, such as soaking, milling, germination, fermentation, autoclave, and cooking. Studies have shown that boiling beans improves their nutritional value by dramatically decreasing the content of lectins [75]. Since lectins may be dissolved in water, exposing them to water (by soaking, for instance) might reduce their concentration. Mung beans, for instance, have a relatively low lectin concentration after being milled and soaked [76,77]. The FDA reports that soaking beans for at least 5 hours and then boiling them in fresh water for at least 30 minutes may entirely eliminate phytohemagglutinin from them. Soaking food before cooking it is an effective technique [78,79]. Boiling the pulses (at 95 °C for 1 h) eliminates nearly all hemagglutinating action. [80]. As previously mentioned, the lectin content of foods can be decreased by various methods of food preparation, which might alter their potential health effects.

### 2.3. Plant Protease İnhibitors

Plant protease inhibitors (PPIs) are a crucial defense mechanism against a broad spectrum of pathogenic microorganisms in plants [81]. They are found in numerous plant parts, but are most prevalent in seeds and tubers, and are triggered in response to insect or disease damage or invasion [82,83]. High amounts of PPIs are frequently found in plants from the Solanaceae, Leguminosae (Fabaceae), and Gramineae (Poaceae) families. Small molecules called plant protease inhibitors suppress proteolytic enzymes. In living organisms, proteases and their inhibitors are found together [84,85].

Depending on structural similarity or sequence homology, more than 6700 plant PPIs may be placed into at least 12 different families [86]. Protein PIs (15 kDa) include serpins, phytocystatins, and Kunitz-type inhibitors (KTI), whereas peptide PIs (15 kDa) include Bowman–Birk inhibitors (BBIs), α-amylase-trypsin inhibitors, mustard-type inhibitors, potato type I and II PIs, and potato metallocarboxypeptidase inhibitors (MCPI). Proteins with a single inhibitory domain constitute the majority of plant PIs. These domains are composed of the following components: compared to undeveloped domains, secondary protein structural elements which are frequently less susceptible to proteolytic degradation (i.e., a-helices or b-sheets); post-translational modifications (i.e., pyroglutamate to protect the N-terminus from aminopeptidases); cyclization (i.e., cyclotides), which shields the peptide termini from both carboxypeptidases and aminopeptidases; and cysteine-stabilization (ex., cystine-knot) [86,87,88,89]. Moreover, the sequence of amino acids, their location, the type of the reactive site or structure, the amount of disulfide bonds, and the catalytic activity are utilized to categorize PPIs [90].

Uncooked grains and legumes, particularly soybeans, typically contain protease inhibitors. In recent years, the biological properties of PPIs, such as their antibacterial, anticoagulant, and antioxidant activity, as well as their ability to inhibit the proliferation of tumor cells, have been observed, indicating their potential application in medicine, agriculture, and technology [91]. Due to the high number of cysteine residues in disulfide bridges, many of these inhibitors are very resistant to chemicals that degrade proteins, heat, pH fluctuations, and proteolysis. The inhibition mechanisms may be classified as follows: based on inhibition through the Michaelis complex, enzyme-substrate complex, and acyl-enzyme complex; through non-productive binds (i.e., inhibitors of apoptosis); and by blocking the active site (i.e., cystatins). There are two types of binding that can occur between PPIs and their targets: reversible and irreversible [91,92,93]. Blocking the active site of an enzyme with this approach inhibits its catalytic activity. In PPIs, the N-terminus, the C-terminus, and the exposed loop are recognized as essential structural features for the inhibition of enzyme function [94].

Plant Pıs’ inhibitors are classified as antinutritional factors due to their capacity to bind to proteases and block their hydrolyzing activity, hence preventing amino acid intake and digestion. In addition, PPIs decrease trypsin and/or chymotrypsin levels by creating inactive complexes with them, so decreasing the quantities of these digestive enzymes. The increased release of trypsin and chymotrypsin can lead to poor protein absorption, the decreased bioavailability of sulfur-containing amino acids (such as methionine and cysteine), slowed growth, muscle mass loss, and pancreatic hypertrophy [93,95,96].

Plant PIs may have several potential health effects. Plant PIs are crucial tools in biotechnology and medicine due to their structure and mode of action [91,97,98]. As a pharmacologically practical approach for proteolysis regulation, the use of protease inhibitors to treat systemic diseases, such as immune, inflammatory, respiratory [98], cardiovascular [99], and neurodegenerative diseases [86], has proved valuable in drug design to inhibit the spread of infections that cause severe diseases, such as acquired immune deficiency syndrome (AIDS) [100], hepatitis [101], SARS-CoV-2 [102], and cancer [103]. These include angiotensin-converting enzyme (ACE) inhibitors for treating hypertension, HIV-1 protease inhibitors for treating AIDS, thrombin inhibitors for treating stroke, and an elastase inhibitor for treating systemic inflammatory response syndrome (SIRS). According to research [104,105], PPIs interact synergistically with antibiotics, increasing their efficacy against antibiotic-resistant microorganisms. In addition, several biological effects, such as anticancer [106], anticoagulant [107], and antioxidant [108] effects, have been shown.

Due to the protein component of their structure, PPIs are vulnerable to heat. Events such as the breakage of covalent bonds, the hydrolysis of peptide bonds, and the exchange or annihilation of disulfide bonds all contribute to the heat degradation of PPIs. Boiling, oven-drying, microwave-baking, and extrusion are popular heat treatments used in households and industries to deactivate, degrade, or reduce the activity of PPIs. In addition, PPIs’ inactivation and sulhydryl/disulfide exchange mechanisms have been linked to protein aggregation and the Maillard reaction [109,110,111].

### 2.4. α-Amylase İnhibitors

Plant seeds are a significant source of α-amylase inhibitors [9]. Numerous plants (cereal grains and legumes) contain so-called α-amylase inhibitors, which regulate the activity of endogenous α-amylase and the immune response to pathogens and parasites. Protease and α-amylase inhibitors function similarly [9]. 

α-amylase is an essential amylase produced by mammals, plants, and microorganisms [112]. This enzyme breaks the α-(1–4)-glycosidic bonds between two adjacent glucose units in amylose, generating glucose, maltose, and oligosaccharides [113]. It has been shown that α-amylase-inhibitors may be beneficial in treating type 2 diabetes [114,115]. An in vitro study has shown that different plants, mainly traditionally used in treating diabetes in Africa or Europe, can inhibit α-amylase. A 90.0% inhibition of α-amylase activity was detected in the extract of Tamarindus indica leaves [116].

### 2.5. Canatoxin-Like Proteins and Ureases

Canatoxin is a toxin first isolated from the seeds of Canavalia ensiformis jack bean [117]. Jack bean seeds contain approximately 0.5% protein in their natural state. Canatoxin is an isoform of the jack bean main seed urease, retaining approximately 30% of the urease’s ureolytic activity [118]. Canatoxin, a neurotoxin, is fatal to rats and mice with an LD_50_ of 2–5 µg/g when administered intraperitoneally; however, the protein is inactive when taken orally because it is unstable at a low pH [117]. Canatoxin induces spinal cord-originated tonic convulsions that result in respiratory distress and, ultimately, animal death [9].

One of the target tissues for canatoxin has been determined to be the central nervous system, and it is possible that certain neurotransmitters will be released in a manner that is both dose- and time-dependent after inoculation with canatoxin [119]. Canatoxin suppresses Ca^2+^ transport by Ca^2+^ ATPase, as demonstrated by experiments involving sarcoplasmic reticulum vesicles. This results in an increase in cytoplasmic Ca^2+^ concentration, which eventually leads to the initiation of exocytosis [120]. Lipoxygenase pathways are likely involved in this toxicity process, as lipoxygenase inhibitors inhibit all of the known toxic effects caused by canatoxin [121]. In addition, it is possible that the hemilectin activity of the canatoxin plays an important part in its association with target cell surfaces and that this helps to explain the tissue-specific toxicity of the toxicity [122].

Ureases are responsible for the conversion of urea to ammonia and carbon dioxide. In the past three decades, novel deleterious properties of ureases, independent of their enzyme activity, have been discovered [123]. Plant ureases are fungitoxic to filamentous fungi and yeasts via a mechanism involving the permeabilization of fungal membranes. There is strong evidence that ureases found in plants and at least some microbes can kill insects [123]. An internal peptide is partially responsible for the entomotoxicity of this compound. This entomotoxicity is dependent on an internal peptide secreted by insect digestive enzymes upon proteolysis of ingested urease. Insects are sensitive to the neurotoxic effects of the total protein and its derived peptides, which impact a variety of other physiological processes, including diuresis, muscular contraction, and immunity. Some ureases cause severe neurotoxicity in animal models when injected; at least some of this toxicity is due to enzyme-independent effects [124]. It has been known for quite some time that bacterial ureases play an essential role in the virulence of illnesses caused by microorganisms that produce urease. Even when their ureolytic activity is inhibited by an irreversible inhibitor, ureases can still stimulate exocytosis in various mammalian cells. This is because they attract eicosanoids and Ca^2+^-dependent pathways [125].

### 2.6. Arcelin

Arcelins are isolated seed proteins in wild bean accessions (*P. vulgaris* L.). The arcelin sequence belong to the arcelin/phytohemagglutinin/a-amylase inhibitor (APA) family, that are all encoded in a single locus known as the APA locus [126]. Arcelins and α-amylase inhibitors share a similar three-dimensional structure as well as a high degree of sequence similarity with lectins, but lack carbohydrate binding sites. α-amylase inhibitors are traditionally regarded as antinutrients that inhibit the assimilation of carbohydrates in livestock diets. However, inhibitor activity produces carbohydrate blockers to control weight gain [127]. Arcelins, conversely, are exclusive to specific wild bean genotypes and may impart seed resistance to phytophagous insects [128]. In addition, all APA proteins are highly resistant to proteolysis by enzymes. Conversely, heat treatment enhances their hydrolysis; however, a residual activity that reduces protein digestibility and toxicity is sometimes detected after cooking [129].

### 2.7. Antimicrobial Peptides

Antimicrobial peptides (AMPs) are molecules that constitute the inherent host defense of numerous organisms, including plants. In addition, AMPs are stated potent immunomodulatory molecules in autoimmune diseases. AMPs appear to perform anti-inflammatory and pro-inflammatory roles in autoimmunity [130]. Recently, there has been a significant increase in interest in the expression of AMPs in plants for three primary reasons: the need for novel approaches in food preservation, plant protection, and the demand for new antimicrobial agents in medicine [131].

AMPs are classified on the basis of sequence and 3D structure similarity, and the similarity in the cysteine motifs, namely the arrangement of cysteines in the polypeptide chain [132]. AMPs are ubiquitous, low-molecular-weight peptides directly targeting microbial pathogens [133]. As the majority of AMPs are cationic, they bind selectively to microbial surfaces. Once they obtain access to the cytoplasmic membrane, they can either disrupt the membrane’s structural integrity or translocate across it to act on intracellular targets [134]. One strategy for preventing food spoilage is to utilize plant AMPs, which have been studied for their potential bioactivities against various human, plant, and food pathogens. As a method of food preservation, the food industry may benefit from developing synthetic AMPs derived from plants with increased bioactivity, improved stability, and decreased cytotoxicity to utilize plant AMPs in diverse food preservation techniques [135]. Additionally, AMPs are useful in helping to develop innovative agricultural plant protection strategies. Disease resistance conferred by AMPs can help overcome losses in yield, quality, and the safety of agricultural crops from plant pathogens.

Plants have various classes of AMPs, including cyclotides, thionins, lipid transfer proteins, snakins, defensins, α-hairpinins, hevein-like peptides, and knottins. In most cases, these bioactive peptides’ biological activity depends upon their binding to the target membrane, followed by membrane permeabilization and disruption. In this study, we reviewed the most widely researched families of thionins and cyclotides [9].

#### 2.7.1. Thionins

Thionins are typically proteins present in various monocot and dicot plants. According to the amino acid sequences and disulfide bond arrangements, thionins are categorized into five structural classes (I-V) [134,136]. Thionins are typically found in higher plants and have potent antibacterial, antifungal, and anticancer properties [135]. Thionins are toxic to numerous biological systems, including bacteria, fungi, and mammalian cell cultures. Thionins have been proposed to play a role in plant defense against pathogen attacks due to their toxicity [9]. Upon pathogen attack, thionin genes are regulated by methyl jasmonate; this plant hormone plays a crucial role in defense reactions. The differential regulation of the thionin loci Thi2.1 and Thi2.2 was observed in *Arabidopsis thaliana*. Thi2.1 expression in flowers is induced by infection with *Fusarium oxysporum* and regulated by methyl jasmonate, whereas Thi2.2 expression in seedlings is independent of jasmonate [136,137]. Thionins exhibit anticancer and cytotoxic effects on mammalian cells. They also exhibit cytotoxic activity that results in hemolysis. Changes in the membrane structure activate endogenous phospholipase-2 and depolarize the membrane, resulting in cell death and anticancer activity [136]. Therefore, these peptides are candidates for the production of novel anticancer medications. In addition, they play a role in seed maturation, dormancy, and germination, in addition to their mobilization during germination [138]. 

#### 2.7.2. Cyclotides

In the 1960s, the initial cyclotide kalata B1 was discovered. According to Gould et al. (2011), Kalata B1 is the primary active component of the Rubiaceae plant *Oldenlandia affinis*, which native people used to create tea to accelerate labor [139]. Cyclotides are plant-derived, small cysteine-rich AMPs. They typically contain 28–37 amino acids and have three characteristic disulfide bonds. They are cyclotides because their peptide bones are cyclized from head to tail. The biological effects of cyclotides include antibacterial, antifungal, insecticidal, and anti-HIV properties and anticancer effects [140]. Consequently, there is a growing interest in utilizing cyclotides, not only for agricultural purposes, but also for drug design in the medical field [141]. One of the first activities described for cyclotides was their ability to perform a hemolytic effect which only occurs in the cyclic condition. When linearized, cyclotides lose their hemolytic activity, indicating that the cyclic backbone is essential for this activity, which also appears to be essential for the other activities of cyclotides [142]. In addition, it was shown that cyclotides had a dose-dependent antiproliferative function on human lymphocytes [143]. Model membrane biophysical investigations have demonstrated that cyclotides can target and disrupt biological membranes. Furthermore, cyclotides bind and insert into lipid membranes [144,145]. Moreover, membrane leakage studies support membrane disruption [146] caused by cyclotides.

### 2.8. Pore-Forming Toxins

Typically, pore-forming toxins (PFTs) are secreted as water-soluble molecules. PFTs are proteins that form water-filled pores in biological membranes. PFTs have been found in bacteria, plants (e.g., Enterolobium contortisiliquum and wheat), fungi, and animals. Enterolobin, a cytolytic protein extracted from the seeds of the tropical tree Enterolobium contortisiliquum, is the most thoroughly researched pore-forming toxin from plants [9].

Numerous pathogens produce pore-forming toxins to attack the host by creating pores in the target cell’s membrane. Typically, pore-forming toxins endure a conformational change and subsequently assemble into an oligomeric structure, which promotes membrane insertion [9]. They associate with the target membrane, form multimers, and endure a conformational change, forming an aqueous pore in the membrane upon recognition and binding to a specific receptor [147]. Different substances, such as ions, small molecules, and large molecules (e.g., proteins) pass through pores of varying sizes [148]. 

The common functions of PFTs include disrupting the function of the epithelium barrier and evading the immunological reactions of the host, both of which contribute to the development and proliferation of bacteria. This group of toxins is an attractive target for the development of novel virulence-targeted therapies that may have comprehensive activity against human pathogens [147]. A growing understanding of the structure and function of PFTs has facilitated the development of biotechnology applications, such as antimicrobial medication development and DNA sequencing [149]. 

## 3. Mechanism of Action

The mechanism of action for each plant toxic protein is reviewed under the relevant heading in detail. A summary of the biological activities of toxic plant proteins is shown in this section and in Table 1. Plant toxic proteins are metabolites and are produced by plants to defend against various hazards (insects, fungi, bacteria) [10]. Toxic or noxious metabolites produced by plants or plant pathogens that adversely affect living organisms are called phytotoxins. There are numerous mechanisms of action of phytotoxins on the physiological processes of the organisms they affect [150]. The fact that plant toxic proteins have such a mechanism of action may cause abnormalities in plant cells, resulting in the plant losing its vitality; on the other hand, the plant may have designed these mechanisms to protect itself and improve its quality of life. [151]. Similarly, in humans, phytotoxins may cause a deterioration in absorption by preventing the functioning of some enzymes, degeneration in cells by interacting with body cells, or various complications (such as vomiting, diarrhea, and poisoning); however, they have positive effects on human health by preventing some adverse mechanisms in the human body or as new agents in the pharmaceutical industry [151,152]. RIPs are toxins that localize to the cell wall after they are produced, reaching the host cytoplasm and inhibiting its ribosomes [153]. Cell surface adhesion—which is essential for colonization, viral infections, bacteria, fungus, and symbiotic microbes associating with the host—is facilitated by lectins. Furthermore, lectins exhibit agglutinating activity [60], which regulate the activity of endogenous α-amylase and the immune response to pathogens and parasites [9]. Canatoxin induces spinal cord-originated tonic convulsions that result in respiratory distress and, ultimately, animal death [9]. Antimicrobial peptides (AMPs) are ubiquitous, low-molecular-weight peptides directly targeting microbial pathogens [133]. Thus, AMPs appear to perform anti-inflammatory and pro-inflammatory roles in autoimmunity [130]. Numerous pathogens produce pore-forming toxins to attack the host by creating pores in the target cell’s membrane [147]. 

## 4. Possible Uses for Plant Toxic Proteins

Plants produce an extensive variety of toxic proteins that are toxic to humans, animals, bacteria, fungi, and viruses. These toxic proteins play a significant role in plant defense, agriculture, and medicine [10,154,155]. Among their positive effects, they ensure the survival and development of plants by improving their defense mechanism [156]. Furthermore, it was discovered that crop yield and product quality improved [155,157].

In agriculture, due to their structural diversity and biological activity, plant toxic proteins are utilized in the development of natural herbicides [155]. Plants have developed natural mechanisms to maintain their normal functioning and metabolic activities by synthesizing toxic proteins in order to defend against environmental stresses, such as extreme salinity, drought, heat, pH, excessive water, the availability of nutrients, plant competition, the abundance of heavy metals, and radiation that is harmful. These natural mechanisms allow plants to maintain their normal functioning and metabolic activities [158].

Several studies have been conducted to exploit the antiviral, antifungal, and insecticidal properties of plant proteins. Using genetic engineering techniques, genes encoding toxic proteins, such as lectins, RIPs, protease inhibitors, and thionines have been transmitted from plants to other plants in order to develop resistance to various pathogens. The objective was to assure improved and more sustainable plant protection in this manner [159,160].

The toxic proitens derived from plants have an essential function in cell cycle regulation, DNA degradation, cytotoxicity, and anticancer effects [161,162]. In particular, RIPs have been utilized to develop immunotoxin-producing medications. β-momorcharin derived from *Momordica charantia* has been demonstrated to be effective against lymphoma, carcinoma, melanoma, cutaneous malignancies, and prostate cancer [163,164]. The U.S. Food and Drug Administration has approved Denileukin diftitox (Ontak), an engineered protein combining interleukin-2 and the diphtheria toxin, for the treatment of cutaneous T-cell lymphoma [165]. Over the past few years, over 450 immunotoxins based on RIPs have been produced and evaluated in cell cultures, animal models, and human patients against a wide range of cancers [166].

**Figure 1:**
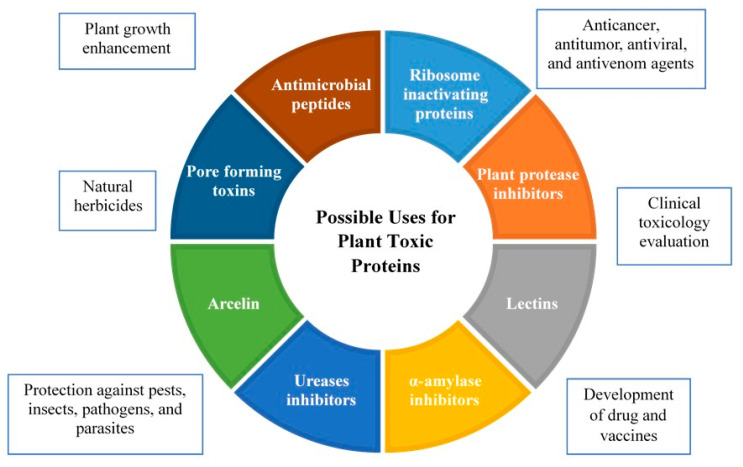
Possible uses for plant toxic proteins (Adapted from [13]).

TCS has been utilized in traditional Chinese medicine to induce abortion and treat hydatidiform moles [167]. The anti-HIV activity of TCS, PAP, and lectins has been demonstrated [168,169]. In addition, α-amylase inhibitors and cyctolotides with their chemical structures and stable properties stand out in drug design and discovery [170]. The possible uses for plant toxic proteins are given in Figure 1.

It has been stated that plant toxic proteins have therapeutic and nourishing effects when consumed by humans in certain quantities [171]. Plant toxic proteins can be eliminated through heating and dehydrating. However, some proteins can tolerate these techniques, so their consumption should not exceed the safe doses [172,173]. The consumption of plant-based products for a sustainable life is increasing day by day [174]. Especially in the food, beverage (tea or plant extracts), and pharmaceutical sectors, the increase in the consumption of plant products increases the consumption of plant toxic proteins It is stated that these toxins can be harmful to human health (especially in children, pregnant, and elderly people) as a result of long-term exposure or accumulation in the body [2]. 

## 5. Removal Strategies of Plant Toxins

Plant toxins are one of the main factors that affect the bioavailability of grain, legume, vegetable, and fruit components. Several factors may lead to micronutrient deficits and mineral deficiency. In addition to lowering the bioavailability of many minerals and nutrients, consuming a diet high in plant toxins can cause toxicity. There are a number of conventional techniques and technologies that may be utilized to lessen plant toxicity. To lower the amount of plant toxins in food, many processing procedures and technologies are employed, such as milling, fermentation, germination, cooking, radiation, autoclaving, chemical treatment, soaking, etc. To decrease the amount of toxins in plants, these approaches can be applied either alone or together [7,175].

Milling is the most conventional technique for separating the bran layer from the grains, hence removing the lectins from the grains. Soaking is one technique used to eliminate water-soluble plant toxins. Soaking reduces cooking time and ensures fermentation and that grains germinate. Autoclaving and cooking are common heat treatment techniques. Due to the high protein content of enzyme inhibitors, they are easily denatured by heat. Fermentation and germination are two of the key processes that decrease the amount of plant toxins in grains and promote in vitro protein digestibility and mineral bioavailability. While gamma radiation may be used to safely remove plant toxins from postharvest maize kernels, genomic technologies can also be employed in effective ways to eliminate RNA pathogens and toxins; however, in vivo testing of genomic technologies has not yet been conducted [96,176].

## 6. Conclusions

The toxic proteins produced by plants can have either beneficial or adverse effects on humans and animals, demonstrating the wide range of their potential applications. Plants have developed a complex defensive mechanism to detect and respond to invading species by synthesizing and storing various chemical and protein-based toxic compounds. Several secondary plant compounds, such as ribosome-inactivating proteins, lectins, plant protease inhibitors, α-amylase inhibitors, canatoxin-like proteins, ureases, arcelin, antimicrobial peptides, and pore-forming toxins, have been discovered. Because of their biological characteristics, these compounds have been utilized by humans in a variety of fields, including natural herbicides, therapeutic/pharmaceutical agents, drug development, and genetic applications in agriculture and medicine. When individuals use or consume significant amounts of plant toxins, particularly children, pregnant women, and older people, mineral absorption and protein digestion can diminish and increase toxicity. Traditional and technological processing methods, such as grinding, soaking, germination, autoclaving, boiling, and fermentation, are used to reduce these proteins’ toxicity and detrimental effects.

To investigate the potential health benefits and biotechnology applications of a protein, it is crucial to have comprehensive knowledge of its structure, variety, biological activity, and mechanism of action. Although substantial progress has been made regarding the biological activity and mechanism of action of toxic proteins, numerous unresolved issues remain, including the possible physiological functions of plants, the potential health benefits, dosage, and new products for use in agriculture and medicine.

## Figures and Tables

**Table 1:** A summary of plant toxic proteins.

Family	Example	Source Plant		Activity	References
Ribosome inactivating proteins					
Type I	Pokeweed antiviral protein, Trichosanthin, Saporin	*Phytolacca americana*, *Trichosanthes kirilowii*, *Saponaria officinalis*	Pokeweed, soapwort	N-glycosidase activity, RNA hydrolases activity, antibacterial, antiviral, antifungal, and insecticidal characteristics activities	[34,36]
Type II	Abrin, Ricin	*Abrus precatorius*, *Ricinus communis*	Rosary pea, castor bean, castor oil		
Type III	b-32, JIP60	*Zea mais*, *Hordeum vulgare*	Maize, barley		
Plant protease inhibitors	Serpins, Phytocystatins, Kunitz-type inhibitors, Bowman-Birk inhibitors, α-amylase-trypsin inhibitors, mustard-type inhibitors, potato metallocarboxypeptidase inhibitors		Chickpea, soybean, barley, sweet potato, lentil, black-eyed pea	Inhibition of proteas hydrolyzing activity, antibacterial, anticoagulant, anticancer, and antioxidant activities	[86,91]
Lectins	Phytohemagglutinin, Lentil lectin, Concanavalin A, Soybean lectin	*Phaseolus vulgaris*, *Vicia faba*, *Canavalia ensiformis*	Lentil, soybean, red and white kidney beans, jack beans	Carbohydrate-binding activity antimicrobial, antibacterial, antifungal, antiviral, and anticancer activities	[59,60]
α-amylase inhibitors	Phaseolin		Cereal grains (wheat, maize, rice, barley), legumes (kidney beans, cowpea, adzuki beans)	Inhibition of α-amylase activity, antihyperglycemic activity	[112,113]
Canatoxin-like proteins and ureases			Mainly in legumes	Ureolytic and pore-forming activity	[124,125]
Arcelin	Arl- 1, Arl-2, Arl-3, Arl-4		Seeds of Phaseolus sp.	N/A	[126,128]
Pore forming toxins	Enterolobin		*Enterolobium contortisiliquum*, wheat	Pore-forming, and antimicrobial activities	[147,148]
Antimicrobial peptides					
Thionins	α/β-thionins, γ-thionins		A number of monocot and dicot plants	Increase in cell membrane permeability, antibacterial and antifungal activities	[135,138]
Cyclotides	Kalata B1		Widely distributed *Rubiaceae plant Oldenlandia affinis*	Pore-forming activity antibacterial, antifungal, insecticidal, and anticancer activities	[140,143]

## Data Availability

Not applicable.
